# Prediction of Re-positivity for Coronavirus Nucleic Acid Among COVID-19 Patients in the Recovery Phase

**DOI:** 10.3389/fmed.2021.620727

**Published:** 2021-05-05

**Authors:** Shu-fen Zhu, Bo Sun, Jin-kuang Li, Yue Cai, Peng-fei Li, Ji-chang Hong, Jin-hai Li, Shi-wen Xu, Xiao-yang Li, Chen-wei Xue, Bin-bin Gu, Jian-fen Wu, Xian-bin Zhou, Hong Suo, Pei-lin Duan, Xin-xin Wu, Shao-wei Li

**Affiliations:** ^1^Department of Critical Care Medicine, The Affiliated Hospital of Inner Mongolia Medical University, Hohhot, China; ^2^Department of Brain Surgery, Zhongxiang People's Hospital, Jinmen, China; ^3^Department of Radiology, Zhongxiang People's Hospital, Jinmen, China; ^4^Department of Gastroenterology, Taizhou Hospital of Zhejiang Province Affiliated to Wenzhou Medical University, Taizhou, China; ^5^Inner Mongolia People's Hospital, Hohhot, China; ^6^Department of Critical Care Medicine, Zhongxiang People's Hospital, Jinmen, China; ^7^Department of Neurology, Zhongxiang People's Hospital, Jinmen, China; ^8^Taizhou Hospital of Zhejiang Province Affiliated to Wenzhou Medical University, Taizhou, China; ^9^Zhongxiang People's Hospital, Jinmen, China; ^10^Department of Respiratory Medicine, The Affiliated Hospital of Inner Mongolia Medical University, Hohhot, China; ^11^Inner Mongolia Medical University, Hohhot, China; ^12^Key Laboratory of Minimally Invasive Techniques & Rapid Rehabilitation of Digestive System Tumor of Zhejiang Province, Taizhou Hospital of Zhejiang Province Affiliated to Wenzhou Medical University, Linhai, China

**Keywords:** COVID-19, re-positivity, virus nucleic acid, risk predication, re-emergence of coronavirus nucleic acids

## Abstract

**Background and Objectives:** Although the pathogenesis and treatment of coronavirus disease 2019 (COVID-19) have been gradually revealed, the risk for re-emergence of coronavirus nucleic acids in recovered patients remains poorly understood. Hence, this study evaluated the risk predictors associated with re-positivity for virus nucleic acid.

**Methods:** Between February 1 and March 20, 2020, we retrospectively reviewed the clinical epidemiological data of 129 COVID-19 patients who were treated at Zhongxiang People's Hospital of Hubei Province in China. Subsequently, a risk prediction model for the re-positivity of virus nucleic acid was developed, and a receiver operating characteristic (ROC) curve was drawn for further validation.

**Results:** In this study, the rate of re-positivity for virus nucleic acid was 17.8% (23/129) where all re-positivity cases were asymptomatic. The median time interval from discharge to nucleic acid re-positivity to discharge after being cured again was 11.5 days (range: 7–23 days). Multivariate logistic regression analysis showed that leukocytopenia [odds ratio (OR) 7.316, 95% confidence interval (CI) 2.319–23.080, *p* = 0.001], prealbumin < 150 mg/L (OR 4.199, 95% CI 1.461–12.071, *p* = 0.008), and hyperpyrexia (body temperature >39°C, OR 4.643, 95% CI 1.426–15.117, *p* = 0.011) were independent risk factors associated with re-positivity. The area under the ROC curve was 0.815 (95% CI, 0.729–0.902).

**Conclusion:** COVID-19 patients with leukocytopenia, low prealbumin level, and hyperpyrexia are more likely to test positive for virus nucleic acid after discharge. Timely and effective treatment and appropriate extension of hospital stays and quarantine periods may be feasible strategies for managing such patients.

## Introduction

In early December 2019, the first case of unexplained coronavirus pneumonia was reported in Wuhan, China (1), which was followed by an outbreak worldwide. In January 2020, Ren et al. (2) led the completion of whole-genome sequencing of the coronavirus and confirmed a homology of more than 85% with bat severe acute respiratory syndrome (SARS)-like coronavirus (bat-SL-CoVZC45). Therefore, the International Virus Classification Committee (ITCV) named it as SARS coronavirus 2 (SARS-CoV-2), which was then officially named as coronavirus disease 2019 (COVID-19) (3). With increasing research being conducted concerning pathogen transmission and mechanisms (4, 5), continuous update of guidelines on treatment and diagnosis (6, 7), and its prevalence has now been controlled and effectively mitigated in China.

Serious dangers concerning the frequent emergence of test re-positivity of virus nucleic acid in recovered COVID-19 patients have been a widespread concern (8–10). Some studies revealed that this rate ranges from 3.3 to 30.8% (9–13). Yuan et al. (13) reported that young patients (<18 years old) had much higher re-positivity rates (30.8%) than those aged ≥18 years (9.5%). However, its mechanism remains unclear and necessitates further research. Of note, most re-positive patients do not show infectivity, which excludes the possibility of simple viral relapse or secondary infection (13–15). A few recent studies have proposed that virology, the detection of specimens, or the patient' s condition might be potential reasons for test re-positivity of virus nucleic acid (16–18).

In this study, we retrospectively analyzed clinical epidemiological data of 129 COVID-19 patients, and evaluated the risk factors associated with re-positivity for virus nucleic acid. Similarly, prompt and effective treatment and appropriate extension of hospitalization and quarantine period may be feasible strategies for patient management.

## Materials and Methods

### Patients

This retrospective study, which complied with the Declaration of Helsinki, was approved by the Ethics Committee of Zhongxiang People's Hospital (ZXRY20200420) and the Ethics Committee of The Affiliated Hospital of Inner Mongolia Medical University (WZ 2020033).

**Figure 1:**
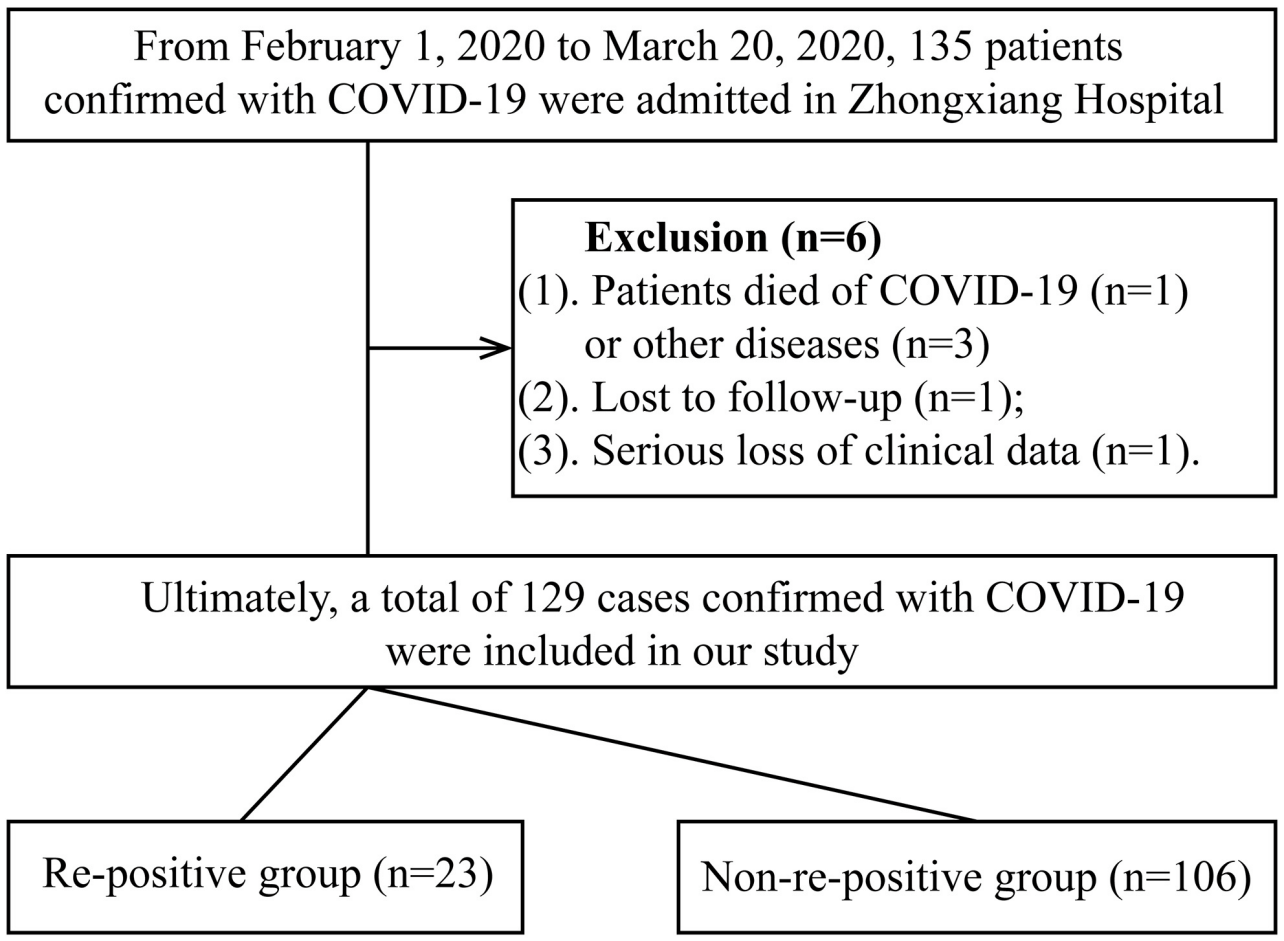
Study flowchart of patient enrollment and grouping. COVID-19, coronavirus disease 2019.

Based on the novel coronavirus pneumonia diagnosis and treatment scheme (Trial Version 4), COVID-19 patients do not only require a clear epidemiological history and clinical manifestations but also must meet at least one of the following conditions: (1) positivity for coronavirus nucleic acid on real-time fluorescent reverse transcription-polymerase chain reaction (RT-PCR); (2) a viral gene sequence showing high homology with the novel coronavirus; and (3) positivity for serum novel coronavirus-specific IgM and IgG antibodies. Furthermore, exclusion criteria were as follows: death due to COVID-19 (*n* = 1) or other diseases [acute cardiovascular disease (*n* = 2), acute renal failure (*n* = 1)], loss to follow-up after being transferred to another hospital (*n* = 1), and serious loss of clinical data (*n* = 1). Finally, 129 COVID-19 cases were included in our study (Figure 1).

### Study Design

Between February 1 and March 20, 2020, we retrospectively analyzed the demographic, clinical, and epidemiological data of 129 COVID-19 patients admitted to our institution. Laboratory findings, radiological results, and therapy course were independently obtained from a prospectively maintained database in Zhongxiang People's Hospital.

Patients were divided into two groups: those with re-positivity (*n* = 23) and without re-positivity (*n* = 106) for the virus nucleic acid group. The differences in sex, age, comorbidities, white blood cell count, body temperature, and prealbumin levels between the two groups were compared, and a risk prediction model was established via multivariate analysis. A receiver operating characteristic (ROC) curve was drawn to validate the model.

Chest computed tomography (CT) results were reviewed by two physicians (Shu-fen Zhu, Pei-lin Duan) and a radiologist (Jin-kuang Li). Leukopenia was defined as a white blood cell count < 4 × 10^9^/L. Hyperpyrexia was defined as body temperature above 39°C. The follow-up outcomes were collected through electronic medical records or telephone interviews by referral physicians or patients with a deadline of until March 20, 2020.

### SARS-CoV-2 Nucleic Acid Testing

RT-PCR was used to detect novel coronavirus nucleic acids. The detection equipment were GeneRotex96 automatic nucleic acid extraction and Gentier96E real-time fluorescent quantitative PCR instruments (Tianlong Technology Co., Ltd., Xi'an, China). The extraction reagent used was the virus DNA/RNA extraction kit (magnetic beads method) (Tianlong Technology Co., Ltd., Xi'an, China). The detection reagent was the SARS-CoV-2 nucleic acid detection kit (PCR probe method) (Da'AN Gene Co., Ltd., Zhongshan, China and Shengxiang Biotechnology Co., Ltd., Hunan, China). The target genes were the ORF1ab and N genes in the SARS-CoV-2 genome, and the positive reading criteria were described in the reagent kit packaging. Specimen sampling, nucleic acid preparation, and amplification were performed strictly using the kit instructions. In case of suspicious results, re-sampling and review were required.

### Clinical Cure Standards for COVID-19 and Re-positivity of Nucleic Acid and Serum Antibody

The standard clinical cure for COVID-19 patients was in reference to the Novel Coronavirus Pneumonia Diagnosis and Treatment Scheme (Trial Version 4): (1) normal body temperature for more than 3 days; (2) significant improvement of respiratory symptoms; (3) notable absorption of inflammation on pulmonary imaging; and (4) two consecutive negative for SARS-CoV-2 nucleic acid tests (sampling interval of at least 24 h). When the patients met these criteria, they were quarantined in a designated area to continue isolation in observation and rehabilitation treatment for at least 14 days. During this period, upper respiratory specimens and blood specimens were collected for a SARS-CoV-2 nucleic acid and serum antibody test on day 14.

### Statistical Analyses

Continuous variables were summarized as mean ± standard deviation (SD) or median plus interquartile range (IQR), and categorical variables were expressed as frequencies and percentages. In the univariate analysis, continuous and categorical variables were assessed via Student's *t*-test (for those with a normal distribution) or Wilcoxon signed-rank test (for those with an abnormal distribution) and chi-squared test, respectively. A *p* < 0.05 was considered statistically significant.

Binary logistic regression was performed to develop the risk prediction model for re-positivity of SARS-CoV-2 nucleic acid, and the ROC curve was used to validate the model. The Statistical Package for the Social Sciences (SPSS, version 23.0; IBM Corp., Armonk, NY, USA) was used for data analysis.

## Results

### Epidemiological History of COVID-19 Patients

A total of 129 COVID-19 patients were retrospectively analyzed. The following findings were gathered: 65 cases (51.9%) with travel or living histories in or around the Wuhan epidemic area; 42 cases (32.6%) with clear contact histories with COVID-19 patients; 10 cases (7.8%) living with COVID-19 patients in the same building, but denying a history of contact; five cases (3.9%) working in the same company as a COVID-19 patient; and five cases (3.9%) with no clear history of contact. Hypertension was found in 16 cases, diabetes in nine cases, chronic cardiovascular disease in nine cases, chronic kidney disease in seven cases, chronic respiratory diseases in six cases [bronchial asthma (*n* = 1), bronchiectasis (*n* = 1), chronic bronchitis (*n* = 1), chronic obstructive pulmonary disease (*n* = 3)], and malignant diseases in five cases (Table 1). The time interval from discharge to re-positivity for coronavirus nucleic acid and serum antibody was 14 days.

**Table 1:** Clinical epidemiological characteristics and a comparative analysis between the re-positivity and non-re-positivity group.

	**Total (*n* = 129)**	**Re-positivity (*n* = 23)**	**Non-re-positivity (106)**	***P*-value**
Age (years), mean ± SD	51.6 ± 14.0	48.9 ± 10.1	52.2 ± 14.7	0.299
Sex, male, *n* (%)	69 (53.5)	11 (47.8)	58 (54.9)	0.176
**Comorbidities**, ***n*** **(%)**				
Diabetes	9 (7.0)	0 (0.0)	9 (8.5)	0.361
Hypertension	16 (12.4)	4 (17.4)	12 (11.3)	0.485
CCD	9 (7.0)	1 (4.3)	8 (7.5)	0.585
CRD	6 (4.7)	1 (4.3)	5 (4.7)	0.939
CKD	7 (5.4)	1 (4.3)	6 (5.7)	0.801
Malignant diseases	5 (3.9)	1 (4.3)	4 (3.8)	0.897
Antibiotics, *n* (%)	92 (71.3)	19 (82.8)	73 (68.9)	0.187
Glucocorticoid, *n* (%)	40 (31.0)	6 (26.1)	34 (32.1)	0.629
Bilateral pneumonia, *n* (%)	83 (64.3)	19 (82.6)	64 (60.4)	0.044
Fever, *n* (%)	87 (67.4)	18 (78.3)	69 (65.1)	0.222
37.3–38.0°C	35 (27.1)	5 (21.7)	30 (28.3)	0.521
38.1–39.0°C	28 (21.7)	4 (17.4)	24 (22.6)	0.782
>39.0°C	24 (18.6)	9 (39.1)	15 (14.2)	0.014
Leukocyte(× 10^9^/L), mean ± SD	4.9 ± 2.0	3.4 ± 0.9	5.3 ± 2.1	< 0.001
Leukopenia, *n* (%)	54 (41.9)	18 (78.3)	36 (34.0)	< 0.001
Neutrophil ratio (%), mean ± SD	65.8 ± 12.3	68.0 ± 7.8	65.3 ± 13.1	0.207
Lymphocyte ratio (%), mean ± SD	23.7 ± 11.0	21.0 ± 8.1	24.2 ± 11.5	0.121
Monocyte ratio (%), mean ± SD	6.3 ± 3.5	7.2 ± 3.2	6.1 ± 3.5	0.163
CRP (mg/L), mean ± SD	33.5 ± 24.5	29.0 ± 13.7	34.9 ± 26.8	0.168
ESR (mm/H), mean ± SD	28.8 ± 29.4	28.9 ± 37.5	28.8 ± 27.5	0.981
PCT (ng/ml), mean ± SD	0.5 ± 0.6	0.3 ± 0.2	0.5 ± 0.7	0.112
LDH (U/L), mean ± SD	215.7 ± 86.3	207.2 ± 48.6	217.3 ± 92.0	0.632
Albumin (g/L), mean ± SD	41.4 ± 21.6	39.7 ± 5.8	41.7 ± 23.7	0.685
Prealbumin (mg/dl), mean ± SD	21.9 ± 11.8	16.1 ± 5.5	23.2 ± 12.4	< 0.001
Prealbumin < 15mg/dl, *n* (%)	38 (29.5)	13 (56.5)	25 (23.6)	0.002
ALT (U/L), mean ± SD	93.3 ± 105.9	76.2 ± 62.0	96.7 ± 112.4	0.430
AST (U/L), mean ± SD	13.6 ± 11.3	14.7 ± 9.4	13.4 ± 11.7	0.620
CK (U/L), mean ± SD	27.3 ± 20.1	28.5 ± 20.6	27.0 ± 20.0	0.747

*SD, standard deviation; CCD, chronic cardiovascular disease; CRD, chronic respiratory disease; CKD, chronic kidney disease; ESR, erythrocyte sedimentation rate*.

*Leukocyte (normal range 4–10); neutrophil ratio (normal range 50–70); lymphocyte ratio (normal range 20–40); monocyte ratio (normal range 3–8); CRP (C-reactive protein; normal range < 4.0); ESR (normal range 0–20); PCT (procalcitonin; normal range 0–0.5); LDH (lactate dehydrogenase; normal range 109–245); prealbumin (normal range 15–40); albumin (normal range 35–55); ALT and AST (alanine aminotransferase and Alanine aminotransferase; normal range 0–40); CK (creatinine kinase; normal range 18–198)*.

### Demographic and Clinical Characteristics of COVID-19 Patients

All 129 patients were diagnosed with COVID-19 based on a positive nasopharyngeal swab nucleic acid test. The rate of re-positivity for virus nucleic acid was 17.8% (23/129) with a mean age of 51.5 ± 14.2 years. The proportion of male (54.3%) and female (45.7%) cases was approximately equal. Pneumonia, suggested by radiography, was detected in 109 cases (84.5%), where 80 (62.0%) of them presented with bilateral pneumonia. There were 87 cases (67.4%) with fever on admission, mainly of low or medium severity. During hospitalization, antiviral drugs (ribavirin, peginterferon, and/or abidor) and immunomodulatory drugs (thymosins) were administered for all COVID-19 patients. Similarly, there were 71 (70.5%) and 40 patients (31.0%) who received antibiotics and glucocorticoids during hospitalization, respectively.

### Predictive Factors Associated With Re-positivity for Virus Nucleic Acid: Findings of Univariate and Multivariate Analyses

The results of the univariate analysis for possible predictive factors associated with the re-emergence of virus nucleic acids are summarized in Table 2. The following four variables were determined to be significant risk factors according to a univariate analysis (*p* < 0.05): leukopenia, prealbumin < 150 mg/L, hyperpyrexia, and bilateral pneumonia. In the binary logistic regression analysis, leukopenia [odds ratio (OR) 7.316, 95% confidence interval (CI) 2.319–23.080, *p* < 0.001], prealbumin < 150 mg/L (OR 4.199, 95% CI 1.461–12.071, *p* = 0.035), and hyperpyrexia (OR 4.643, 95% CI 1.426–15.117, *p* = 0.035) were independent risk predictors associated with re-positivity for SARS-CoV-2 RNA.

**Table 2:** Predictive factors associated with re-positivity for coronavirus nucleic acid in a multivariate analysis.

	**OR**	**95% CI**	***P*-value**
Leukopenia	7.316	2.319–23.080	0.001
Hyperpyrexia	4.643	1.426–15.117	0.011
Prealbumin < 15mg/dl	4.199	1.461–12.071	0.008

*OR, odd ratio; CI, confidence interval*.

**Figure 2:**
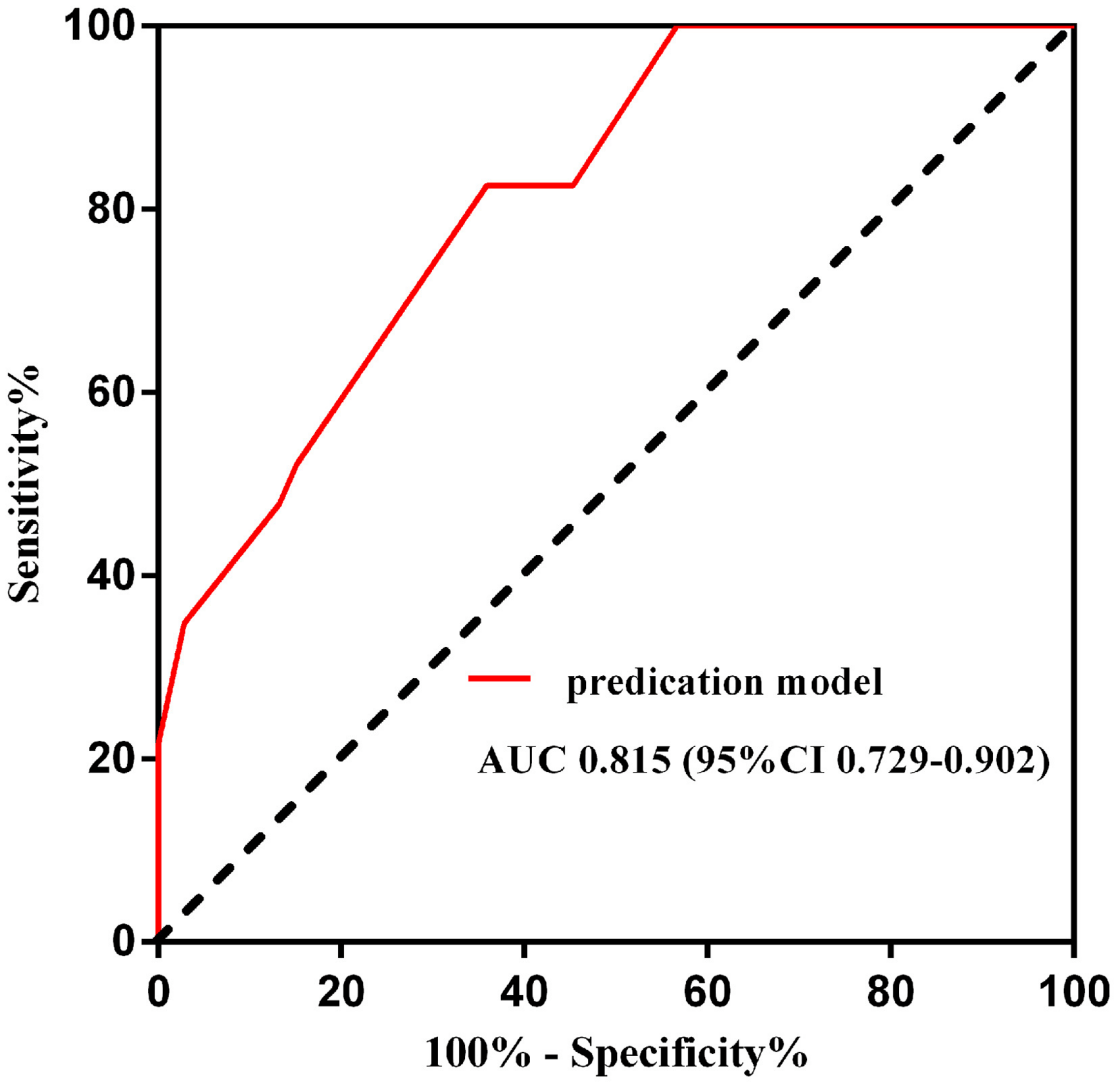
Receiver operating curve of the predication model for NCR. AUC, area under the curve; CI, confidence interval.

On this prediction model, an ROC curve was drawn to validate the model. The area under the ROC curve was 0.815 (95% CI 0.729–0.902), which suggested that the model had moderate to good predictive accuracy (Figure 2).

### Clinical Outcomes After Re-positivity for Coronavirus Nucleic Acid

All re-positive patients were asymptomatic, and the emergence of new pulmonary infiltration or consolidation was not revealed on chest CT. Among the 23 re-positive cases, five were discovered during a community medical examination after isolation had been lifted. The family members of these five patients were also quarantined, and all coronavirus nucleic acid tests were found to be negative during this period. These patients were transferred to the hospital for a second period of 14 days, in which RT-PCR of the blood, nasopharyngeal swabs, and anal swabs were performed on days 1, 4, 7, and 14. Furthermore, the coronavirus nucleic acid tests of five patients turned negative on day 4, 10 patients turned negative on day 7, and eight patients turned negative on day 14.

## Discussion

Most COVID-19 patients had a favorable prognosis under “Management of Diagnosis and Treatment of Novel Coronavirus Pneumonia Scheme Trial Version 4.” However, the emergence of test re-positivity of SARS-CoV-2 nucleic acid in recovered patients will exacerbate the global situation.

A large study in South Korea showed that 292 (3.3%) out of 8,922 recovered COVID-19 patients showed re-positivity of virus nucleic acid post-discharge, although there was no detailed description concerning whether all of the recovered patients had been tested or whether only the symptomatic ones had been tested after discharge (11). Yuan et al. (13) reported that the re-positivity rate of patients <18 years old (30.8%) was much higher than that of patients ≥18 years old (9.5%). In this study, the re-positivity rate was 17.8% (23/129), which was close to the average level reported in previous studies (range: ~14.5–16.7%) (9, 10, 12, 14). To avoid false negatives as much as possible, all patients (*n* = 129) underwent two consecutive SARS-CoV-2 nucleic acid tests (sampling interval of at least 24 h) before discharge.

Hyperexia and low serum prealbumin levels were determined to be independent risk factors associated with test re-positivity of SARS-CoV-2 RNA. Similarly, He et al. (19) reported that the severity of cytokine inflammatory storm was directly related to the severity of disease, as the long-term maintenance of a body temperature above 39°C (hyperpyrexia) is a symptom of serious infection. Mahallawi et al. (20) further demonstrated a remarkable pro-inflammatory cytokine response during the acute phase of human MERS-CoV infection. The expression of IFN-γ, TNF-α, IL-15, and IL-17 secreted by pro-inflammatory Th1 and Th17 cells differed significantly between patients with and without this infection. Thus, hyperpyrexia leading to an increased risk of re-positivity of SARS-CoV-2 nucleic acid may be achieved by inducing a cytokine inflammatory storm. Regarding the role of prealbumin in re-positive patients, it was an acute negative time reactive protein similar to albumin, of which the level was significantly lower in COVID-19 patients with a poor prognosis than in those with a good prognosis (21). More time may be required for patients with low serum prealbumin levels to completely eliminate SARS-CoV-2. This may somehow explain why a low serum prealbumin level was associated with test re-positivity for nucleic acid.

Leukopenia was another independent risk factor for re-positivity for nucleic acid. Guo et al. (22) indicated that leukocytes, especially lymphocytes (CD3+, CD4+, and CD8+), were significantly reduced in patients who died of viral pneumonia compared with survivors. Changes in the peripheral blood leukocyte count and lymphocyte subsets may play an important role in the pathogenesis of SARS-CoV-2 infection (23). Furthermore, several related studies have also shown that compared with patients with mild COVID-19 infection, the memorability and cytotoxicity of CD8+T cells in severe patients had significantly reduced (1, 20, 24). Lymphocyte apoptosis, immunological injury, and bone marrow suppression may be critical mechanisms leading to lymphopenia observed in severe COVID-19 cases (25, 26). However, few studies have demonstrated a relationship between lymphopenia and re-positivity for coronavirus nucleic acid. A comprehensive analysis of the results showed that the clinical manifestations of hyperpyrexia, leukopenia, and low prealbumin levels indicated the tendency for severe SARS-CoV-2 infection. The lymphocyte count in patients with re-positivity was lower than those without re-positivity, although not to a significant degree. This may have been due to the small sample size; hence, large-scale multicenter trials are suggested.

Since all discharged patients followed strict self-isolation protocols, reinfection was relatively unlikely to have led to re-positivity for SARS-CoV2 nucleic acid. All patients with re-positivity were asymptomatic and showed no signs of new pulmonary infiltration on chest CT. Furthermore, none presented with infectivity, and nearly all patients' viral nucleic acid tests turned negative again within a relatively short period (14). The causes of re-positivity of viral nuclear acid might be a false negative nucleic acid test at the time of discharge, or the viral load after treatment is below the lower limit of nucleic acid test. During the isolation period after discharge from the hospital, the viral load increased again, and the nucleic acid re-positivity occurred. Additionally, a recent study (13) suggested that re-positivity for SARS-CoV-2 RNA might be considered a process of virus shedding, so the re-positive patients were not infectious. However, different findings were shown in several case reports (27, 28), where infectivity was still detected in re-positive patients who had shown multiple negative nasopharyngeal swab tests. Therefore, as there is no clear evidence that re-positive patients cannot transmit the disease, these patients should be followed up scientifically and strictly isolated after discharge to avoid the risk of disease transmission.

However, several limitations must be considered as well. First, this study was retrospective in nature, which is inevitably susceptible for selective bias, observational bias, and confounding bias. Prospective studies concerning COVID-19 are necessary, and propensity score matching (PSM) might be a feasible way to balance out the biases in a retrospective study. Second, this study was conducted at a single center, and the small sample size makes it difficult to generalize the results. Furthermore, we preliminarily demonstrated that leukopenia was an independent risk factor related to re-positivity for SARS-CoV-2 nucleic acid and have yet to subdivide this factor into specific types of white blood cells, such as lymphocytes and neutrophils. To address these shortcomings, further multi-center, large-scale studies are needed.

Immunoglobulin G (IgG) and immunoglobulin M (IgM) are two specific serum antibodies for SARS-CoV-2 infection. As the main antibody during the humoral immunity, IgG has a high affinity for the pathogens and is widely distributed in the body, which is also the main force of the body to fight infection. The peak of IgG secretion appears later in infection, but it lasts for a long time. Therefore, IgG is commonly regarded as the main antibody for serological diagnosis and monitoring after vaccination. However, IgM appears in the early stage of pathogen infection and disappears shortly after acute infection (29). In this study, due to the limited conditions of serum antibody at the early stage of COVID-19 pandemic, viral antibody tests were only performed in a part of re-positive cases (*n* = 15), who were older than other re-positive patients (details in Table 3). The serum antibodies (IgG and IgM) of all re-positive patients were significantly increased, especially those of IgG, which indicated that those re-positive patients were in the recovery stage. Moreover, in the follow-up epidemiological investigation, it was found that after all COVID-19 patients (*n* = 129) were cured and discharged from the hospital, no new cases of COVID-19 were reported in Zhongxiang City.

**Table 3:** Antibody detection of SARS-CoV-2 viral RNA re-positive patients.

	**Sex**	**Age (years)**	**COVID-19**	**COVID-19**
			**IgG+IgM (S/CO)**	**IgM (S/CO)**
Case 1	Male	68	26.06	1.35
Case 2	Female	35	25.51	1.31
Case 3	Male	30	138.61	6.35
Case 4	Female	44	5.61	**0.44**
Case 5	Male	48	82.03	11.70
Case 6	Male	58	46.10	1.29
Case 7	Female	56	122.27	2.78
Case 8	Female	61	28.98	1.14
Case 9	Male	50	32.49	1.22
Case 10	Female	52	39.95	**0.27**
Case 11	Female	38	169.28	6.53
Case 12	Female	43	89.83	16.91
Case 13	Male	43	4.03	**0.23**
Case 14	Female	48	21.14	**0.63**
Case 15	Male	52	27.15	4.12

*IgM, immunoglobulin M; IgG, immunoglobulin G; S/CO, sample/cut off; S/CO < 1 was a negative result; S/CO ≥ 1 was a positive result for the antibody (IgG+IgM and IgM). Bold indicates results that are S/CO < 1*.

In summary, COVID-19 patients with leukopenia, low serum prealbumin levels, and hyperpyrexia are more likely to show re-positivity for coronavirus nucleic acid after discharge than others. Although this study was retrospective, single center (Zhongxiang People's Hospital), and had a small sample size (*n* = 129), but with timely and effective treatment, the appropriate extension of hospitalization and the quarantine period may be feasible strategies for managing such patients.

## Data Availability Statement

The raw data supporting the conclusions of this article will be made available by the authors, without undue reservation.

## Ethics Statement

The studies involving human participants were reviewed and approved by Ethics Committee of Zhongxiang People's Hospital. Written informed consent to participate in this study was provided by the participants' legal guardian/next of kin.

## Author Contributions

S-fZ, BS, J-kL, J-cH, J-hL, X-yL, C-wX, HS, and P-lD participated in the clinical treatment. YC, S-wX, X-xW, and S-wL wrote the original draft. P-fL, B-bG, and J-fW undertook validation, writing, review, and editing. All authors contributed to the article and approved the submitted version.

## Conflict of Interest

The authors declare that the research was conducted in the absence of any commercial or financial relationships that could be construed as a potential conflict of interest.

## References

[1] Lu R Zhao X Li J Niu P Yang B Wu H. Genomic characterisation and epidemiology of 2019 novel coronavirus: implications for virus origins and receptor binding. Lancet. (2020) 395:565–74. 10.1016/S0140-6736(20)30251-8 32007145 PMC7159086

[2] Ren LL Wang YM Wu ZQ Xiang ZC Guo L Xu T. Identification of a novel coronavirus causing severe pneumonia in human: a descriptive study. Chin Med J (Engl). (2020) 133:1015–24. 10.1097/CM9.0000000000000722 32004165 PMC7147275

[3] The species Severe acute respiratory syndrome-related coronavirus: classifying 2019-nCoV and naming it SARS-CoV-2. Nat Microbiol. (2020) 5:536–44. 10.1038/s41564-020-0695-z 32123347 PMC7095448

[4] Chen N Zhou M Dong X Qu J Gong F Han Y. Epidemiological and clinical characteristics of 99 cases of 2019 novel coronavirus pneumonia in Wuhan, China: a descriptive study. Lancet. (2020) 395:507–13. 10.1016/S0140-6736(20)30211-7 32007143 PMC7135076

[5] Clinical findings in a group of patients infected with the 2019 novel coronavirus (SARS-Cov-2) outside of Wuhan China: retrospective case series. BMJ. (2020) 368:m792. 10.1136/bmj.m792 32075786 PMC7224340

[6] Kobayashi T Masumoto J Tada T Nomiyama T Hongo K Nakayama J. Prognostic significance of the immunohistochemical staining of cleaved caspase-3, an activated form of caspase-3, in gliomas. Clin Cancer Res. (2007) 13:3868–74. 10.1158/1078-0432.CCR-06-2730 17606719

[7] Zhou X Yang W Li J. Ca2+- and protein kinase C-dependent signaling pathway for nuclear factor-κB activation, inducible nitric-oxide synthase expression, and tumor necrosis factor-alpha production in lipopolysaccharide-stimulated rat peritoneal macrophages. J Biol Chem. (2006) 281:31337–47. 10.1074/jbc.M602739200 16923814

[8] Lan L Xu D Ye G Xia C Wang S Li Y. Positive RT-PCR test results in patients recovered from COVID-19. JAMA. (2020) 323:1502–03. 10.1001/jama.2020.2783 32105304 PMC7047852

[9] Xing Y Mo P Xiao Y Zhao O Zhang Y Wang F. Post-discharge surveillance and positive virus detection in two medical staff recovered from coronavirus disease 2019 (COVID-19), China, January to February 2020. Euro Surveill. (2020) 25:2000191. 10.2807/1560-7917.ES.2020.25.10.2000191 32183934 PMC7078824

[10] Qu YM Kang EM Cong HY. Positive result of Sars-Cov-2 in sputum from a cured patient with COVID-19. Travel Med Infect Dis. (2020) 34:101619. 10.1016/j.tmaid.2020.101619 32160971 PMC7129439

[11] Kang YJ South Korea's COVID-19 infection status: from the perspective of re-positive test results after viral clearance evidenced by negative test results. Disaster Med Public Health Prep. (2020) 14:762–3. 10.1017/dmp.2020.168 32438941 PMC7298089

[12] Yuan J Kou S Liang Y Zeng J Pan Y Liu L. PCR assays turned positive in 25 discharged COVID-19 patients. Clin Infect Dis. (2020) 714:2230–2. 10.1093/cid/ciaa398 PMC7184423 32266381

[13] Yuan B Liu HQ Yang ZR Chen YX Liu ZY Zhang K. Recurrence of positive SARS-CoV-2 viral RNA in recovered COVID-19 patients during medical isolation observation. Sci Rep. (2020) 10:11887. 10.1038/s41598-020-68782-w 32681141 PMC7368008

[14] Wong J Koh WC Momin RN Alikhan MF Fadillah N Naing L. Probable causes and risk factors for positive SARS-CoV-2 test in recovered patients: evidence from Brunei Darussalam. J Med Virol. (2020) 92:2847–51. 10.1101/2020.04.30.20086082 32558947 PMC7323238

[15] Zhu H Fu L Jin Y Shao J Zhang S Zheng N. Clinical features of COVID-19 convalescent patients with re-positive nucleic acid detection. J Clin Lab Anal. (2020) 34:e23392. 10.1002/jcla.23392 32506726 PMC7300578

[16] Zhang T Cui X Zhao X Wang J Zheng J Zheng G. Detectable SARS-CoV-2 viral RNA in feces of three children during recovery period of COVID-19 pneumonia. J Med Virol. (2020) 92:909–14. 10.1002/jmv.25795 32222992 PMC7228213

[17] Zhu N Zhang D Wang W Li X Yang B Song J. A Novel Coronavirus from patients with pneumonia in China, 2019. N Engl J Med. (2020) 382:727–33. 10.1056/NEJMoa2001017 31978945 PMC7092803

[18] Wu A Peng Y Huang B Ding X Wang X Niu P. Genome composition and divergence of the novel coronavirus (2019-nCoV) originating in China. Cell Host Microbe. (2020) 27:325–8. 10.1016/j.chom.2020.02.001 32035028 PMC7154514

[19] He L Ding Y Zhang Q Che X He Y Shen H. Expression of elevated levels of pro-inflammatory cytokines in SARS-CoV-infected ACE2+ cells in SARS patients: relation to the acute lung injury and pathogenesis of SARS. J Pathol. (2006) 210:288–97. 10.1002/path.2067 17031779 PMC7167655

[20] Mahallawi WH Khabour OF Zhang Q Makhdoum HM Suliman BA. MERS-CoV infection in humans is associated with a pro-inflammatory Th1 and Th17 cytokine profile. Cytokine. (2018) 104:8–13. 10.1016/j.cyto.2018.01.025 29414327 PMC7129230

[21] Liu W Tao ZW Wang L Yuan ML Liu K Zhou L. Analysis of factors associated with disease outcomes in hospitalized patients with 2019 novel coronavirus disease. Chin Med J (Engl). (2020) 133:1032–8. 10.1097/CM9.0000000000000775 32118640 PMC7147279

[22] Guo L Wei D Zhang X Wu Y Li Q Zhou M. Clinical features predicting mortality risk in patients with viral pneumonia: the MuLBSTA score. Front Microbiol. (2019) 10:2752. 10.3389/fmicb.2019.02752 31849894 PMC6901688

[23] Wang F Nie J Wang H Zhao Q Xiong Y Deng L. Characteristics of peripheral lymphocyte subset alteration in COVID-19 pneumonia. J Infect Dis. (2020) 221:1762–9. 10.1093/infdis/jiaa150 32227123 PMC7184346

[24] Xu Z Shi L Wang Y Zhang J Huang L Zhang C. Pathological findings of COVID-19 associated with acute respiratory distress syndrome. Lancet Respir Med. (2020) 8:420–2. 10.1016/S2213-2600(20)30076-X 32085846 PMC7164771

[25] Meyre PB Radosavac M Baumann L Piso RJ Hoffmann M. COVID-19 in a patient with accidental drug-induced neutropenia. Eur J Case Rep Intern Med. (2020) 7:001848. 10.12890/2020_001848 32908840 PMC7473687

[26] Chen RF Chang JC Yeh WT Lee CH Liu JW Eng HL. Role of vascular cell adhesion molecules and leukocyte apoptosis in the lymphopenia and thrombocytopenia of patients with severe acute respiratory syndrome (SARS). Microbes Infect. (2006) 8:122–7. 10.1016/j.micinf.2005.06.007 16182592 PMC7110783

[27] Zhang JF Yan K Ye HH Lin J Zheng JJ Cai T. SARS-CoV-2 turned positive in a discharged patient with COVID-19 arouses concern regarding the present standards for discharge. Int J Infect Dis. (2020) 97:212–4. 10.1016/j.ijid.2020.03.007 32200109 PMC7270599

[28] Bentivegna E Sentimentale A Luciani M Speranza ML Guerritore L Martelletti P. New IgM seroconversion and positive RT-PCR test after exposure to the virus in recovered COVID-19 patient. J Med Virol. (2021) 93:97–8. 10.1002/jmv.26160 32525558 PMC7300757

[29] Paterson RW Brown RL Benjamin L Nortley R Wiethoff S Bharucha T. The emerging spectrum of COVID-19 neurology: clinical, radiological and laboratory findings. Brain. (2020) 143:3104–20. 10.1093/brain/awaa240 32637987 PMC7454352

